# Diabetes and CVD risk during angiotensin-converting enzyme inhibitor or angiotensin II receptor blocker treatment in hypertension: a study of 15 990 patients

**DOI:** 10.1038/jhh.2014.43

**Published:** 2014-06-26

**Authors:** L P Hasvold, J Bodegård, M Thuresson, J Stålhammar, N Hammar, J Sundström, D Russell, S E Kjeldsen

**Affiliations:** 1 The Faculty of Medicine, University of Oslo, Oslo, Norway; 2 AstraZeneca, Nordic-Baltic, Norway; 3 Department of Cardiology, Ullevaal Hospital, Oslo, Norway; 4 Statisticon AB, Uppsala, Sweden; 5 Department of Public Health and Caring Sciences, Uppsala University, Uppsala, Sweden; 6 Institute of Environmental Medicine, Karolinska Institutet, Stockholm, Sweden; 7 AstraZeneca R&D, Mölndal, Sweden; 8 Department of Medical Sciences and Uppsala Clinical Research Center, Uppsala University, Uppsala, Sweden; 9 Department of Neurology, Rikshospitalet, University of Oslo, Oslo, Norway; 10 Department of Cardiology, Ullevaal Hospital, University of Oslo, Oslo, Norway

## Abstract

Differences in clinical effectiveness between angiotensin-converting enzyme inhibitors (ACEis) and angiotensin receptor blockers (ARBs) in the primary treatment of hypertension are unknown. The aim of this retrospective cohort study was to assess the prevention of type 2 diabetes and cardiovascular disease (CVD) in patients treated with ARBs or ACEis. Patients initiated on enalapril or candesartan treatment in 71 Swedish primary care centers between 1999 and 2007 were included. Medical records data were extracted and linked with nationwide hospital discharge and cause of death registers. The 11 725 patients initiated on enalapril and 4265 on candesartan had similar baseline characteristics. During a mean follow-up of 1.84 years, 36 482 patient-years, the risk of new diabetes onset was lower in the candesartan group (hazard ratio (HR) 0.81, 95% confidence interval (CI) 0.69–0.96, *P*=0.01) compared with the enalapril group. No difference between the groups was observed in CVD risk (HR 0.99, 95% CI 0.87–1.13, *P*=0.86). More patients discontinued treatment in the enalapril group (38.1%) vs the candesartan group (27.2%). In a clinical setting, patients initiated on candesartan treatment had a lower risk of new-onset type 2 diabetes and lower rates of drug discontinuation compared with patients initiated on enalapril. No differences in CVD risk were observed.

## Introduction

The renin–angiotensin system is targeted by two of the most widely used antihypertensive medication classes: angiotensin-converting enzyme inhibitors (ACEis) and angiotensin receptor blockers (ARBs). ACEis and ARBs inhibit the renin–angiotensin system differently and may therefore differ in their preventive effects against both diabetes and cardiovascular disease (CVD).

ACEis and ARBs have been reported to be associated with a reduced onset of type 2 diabetes compared with placebo and other antihypertensive treatments.^1, 2, 3, 4^ A meta-analysis by Elliot and Meyer^5^ demonstrated a lower risk of type 2 diabetes in patients treated with ARBs compared with ACEis. Possible explanations for this is the different effects of these medications on glucose metabolism through activation of different parts of the PPAR (peroxisome proliferator-activated receptors) system or more effective blockade of angiotensin type I receptors and the subsequent development of vascular insulin resistance and impaired endothelial nitric oxide-mediated relaxation.^6, 7^ However, no direct comparisons between ACEis and ABRs regarding risk of new-onset diabetes has previously been reported in patients with hypertension.

A few studies have compared the preventive effects of treatment with ACEis vs ARBs on CVD in high CV risk patients with neutral results.^8, 9^ Potential differences in the preventive effects of these drugs on CVD outcomes in uncomplicated hypertension patients are yet unknown.

Candesartan, being one of the two most frequently prescribed ARB in Sweden was chosen to represent the ARBs in this comparison in order to reduce potential confounding. Candesartan was also shown to be more effective in reducing CVD than losartan, the other most commonly used ARB in Sweden.^10^ Enalapril was chosen to represent the ACEis because of identical indications to candesartan and being the most frequently prescribed ACEi in Sweden (75% of patients receiving ACEis).

The aim of the study was to investigate differences in the risk for new-onset type 2 diabetes and CVD in patients initiated on antihypertensive treatment with enalapril or candesartan.

## Patients and methods

The study protocol was reviewed and approved by the Regional Research Ethics Committee in Uppsala, Sweden and registered with ClinicalTrials.gov, number NCT01152567.

Sweden has a tax-funded health-care system, providing primary and secondary care without out-of-pocket expenses and reimbursement for all prescribed drugs for chronic diseases, including hypertension. Patients are normally followed by a general practitioner.

### Study population

Men and women with hypertension identified at 71 primary care centers from 1 January 1999 to 31 December 2007, aged ⩾18 years, who were prescribed for the first time either enalapril (Anatomical Therapeutic Chemical (ATC): C09A A02 or C09B A02) or candesartan (ATC: C09 CA06 or C09 DA06), with or without a fixed combination with hydrochlorothiazide, were eligible for the study. The first prescription of the study drug within the study period was defined as the start of the study. Exclusion criteria were a recorded diagnose of CVD, diabetes, chronic kidney disease or malignancy (data in Supplementary Table S1). Patients who were prescribed vitamin K antagonists, clopidogrel, acetylic salicylic acid, digitalis glycosides, aldosterone antagonists, loop diuretics, nitrates or anti-diabetes drugs within 15 months before the start of the study were considered to have potential CVD or diabetes and were excluded.

Data were extracted from the primary medical records at the primary care centers using an established software system.^11^ Morbidity before and after the start date of the study was collected from the National Patient Register, inpatient (admission and discharge dates and main and secondary diagnoses) and outpatient hospital care.^12^ Mortality during the follow-up was ascertained using the National Cause of Death register (date and cause(s) of death). Data regarding socio-economic status (educational level) were collected from the national censuses at Statistics Sweden. The linkage of data obtained from the national registers and primary care centers was performed by the Swedish National Board of Health and Welfare. Social security numbers, used to identify included patients in all health-care contacts, were replaced with study ID numbers before further data processing.

An attempt was made in the recruitment of study sites to ensure a representative selection of primary care centers in Sweden: a mix of rural and urban areas; public and private care providers; and small, mid-sized, and large primary care centers (data in Supplementary Table S2).The study sample represents approximately 7% of the total number of the primary care centers in Sweden.

### Baseline examinations

Data on age, gender, blood pressure values and body mass index, laboratory/blood samples, diagnoses according to International Classification of Diseases, 9 and 10th revision, Clinical Modification (ICD-9/10-CM) codes, number of visits and prescribed drugs were extracted from the primary care journals. The baseline for the blood pressure value was calculated as the mean of the last three measurements during the time period 15 months before until 14 days after the start of enalapril or candesartan treatment. Blood pressure at 6 months was calculated as the mean of measurements 2 weeks to 6 months after the start of the study. From 12 months and onwards, 6-monthly blood pressures were calculated as the mean of measurements from 6 months before to 6 months after the specific time point.

### Follow-up and outcomes

Patients were eligible for analysis while they remained on study drug treatment. The observation period ended on the date when the patient died, discontinued the study drug treatment, started a new C09-medication/renin–angiotensin system inhibiting drug or on 31 December 2007.

The criteria for the diagnosis of diabetes in Sweden is normally based on elevated plasma glucose values (>7.0 mmol l^−1^) and/or a positive oral glucose tolerance test. The end point for diabetes was a recorded primary care or hospital discharge diagnosis of type 2 diabetes (ICD-9 code 250, ICD-10 codes E10-E14) and/or prescription of a drug within the ATC system class A10. This end point for diabetes diagnosis have been validated in other studies.^13^ The end point for assessing CVD consisted of a recorded diagnosis of all non-fatal and fatal CVD (myocardial infarction, unstable angina, chronic ischemic heart disease, peripheral artery disease, heart failure, cardiac arrhythmias and stroke) as defined by ICD codes (see Supplementary Table S1).^10^

### Statistical methods

The study database was owned and managed by the Department of Public Health and Caring Sciences, Uppsala University, Uppsala, Sweden. The data were processed and analyzed by an independent statistical contract company (Statisticon AB, Stockholm, Sweden).

All descriptive data are given as mean (s.d.) or percentage (%). Time to event end points were analyzed using the Cox proportional hazards regression models, and the results are presented as hazard ratios (HRs) with 95% confidence intervals (CIs) and corresponding *P*-values. If one patient had several end points, only the first was used in the survival model. Time to diabetes or CVD was analyzed separately.

### Selection of covariates for the primary analysis

The main analysis is an adjusted model with adjustment for age and gender at baseline, socio-economic status and year of the start of the study. Patients with a history of renal disease, CVD and/or diabetes were excluded from this study. Age, gender, elevated blood glucose, overweight and low socio-economic status are known risk factors for diabetes.^14, 15, 16^ High cholesterol and hypertension are additionally known risk factors for CVD.^17^

All included patients had hypertension, and there was no difference between the two treatment groups regarding baseline lipid values and statin use. The socio-economic status is associated with smoking pattern, overweight and physical activity, thus a risk factor for diabetes and CVD.^16, 18^ The treatment patterns (diagnoses, treatment targets) may change over time, and year of the start of the study was included as covariate.

The main analysis was supported by sensitivity analyses where additional covariates with incomplete coverage at baseline were included and analyses with exclusion of end points recorded within a specific time frame after the start of the study. Furthermore, for a complementary analysis, propensity scores were estimated corresponding to the probability of receiving the treatment given the baseline covariates. A matched propensity score analysis was performed in order to address confounding associated with the indication for treatment.^19^

### Sensitivity analyses diabetes

For diabetes, additional sensitivity analyses were performed where baseline hemoglobin A1c (HbA1c), blood glucose and body mass index were included as additional covariates. The number and percentage of patients with high HbA1c (>7.0%) or blood glucose (>7.0 and >10.0 mmol l^−1^) values at baseline was also estimated. Analyses were performed where patients with high baseline HbA1c and blood glucose values were excluded. The diagnosis of diabetes within 6 and 12 months after the start of the study were also excluded in extra analyses for diabetes and CVD.

### Sensitivity analyses for diabetes and CVD

Propensity score methods have become widely used tools for confounding control in non-randomized studies of drug effectiveness.^19, 20^ The propensity scores for receiving either enalapril or candesartan were calculated using a logistic regression model in which the dependent variable was use of enalapril or candesartan. Independent covariates included in the model were gender, age, year of the start of the study, systolic blood pressure, total cholesterol, blood glucose, socio-economic status, beta blockers, statins, calcium antagonists and thiazides as covariates. Blood glucose was selected as a covariate for laboratory samples related to diabetes, as the elevated blood glucose is the main diagnostic criterion for diabetes in Sweden. The resulting propensity scores were matched pair wise using callipers of width equal to 0.2 of the s.d. of the propensity score using the matching package in R.^21, 22^ Risk of new-onset diabetes and CVD were calculated using a Cox proportional hazards model stratified by the matched pairs.

For both end points, the same model for adjusted Cox regression with multiple imputation of systolic blood pressure as additional covariate was applied. The potential effect of variation in proportion of included patients per year in the two cohorts was also studied by analyzing the cohorts of patients included before and after 2005 separately. The presented *P*-values are not adjusted for multiplicity, and thus in the interpretation of the results one should take the total number of comparisons into account.

## Results

**Figure 1:**
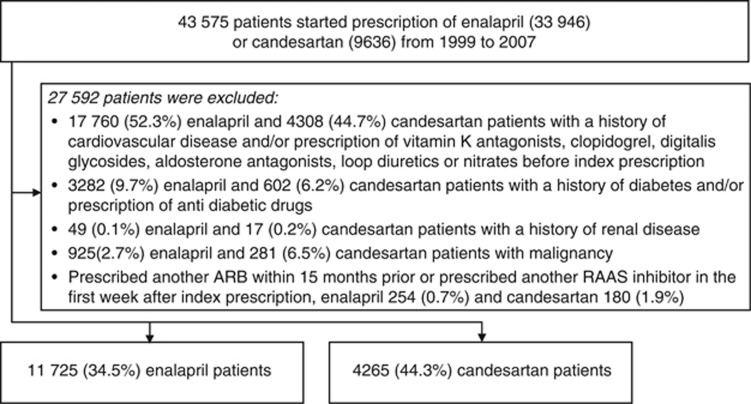
Patient flow.

Of the 43 576 eligible patients; 33 946 (77.9%) were prescribed enalapril and 9636 (22.1%) candesartan. In the 27 592 patients with exclusion criteria, 66% (*n*=22 221) were excluded in the enalapril group and 56% (*n*=5371) in the candesartan group (Figure 1). The remaining study population consisted of 15 990 patients; 11 725 treated with enalapril and 4265 with candesartan. All 71 primary care centers prescribed both enalapril and candesartan, although in various ratios.

### Baseline characteristics

The baseline characteristics for the included patients are summarized in Table 1. Compared with the candesartan patients, enalapril patients were slightly older (+1.0 years), less frequently females (−4%), had a higher systolic blood pressure (+0.1 mm Hg), higher blood glucose (+0.1 mmol l^−1^), higher HbA1c (+0.2%) and lower serum creatinine (2.6 μmol l^−1^). Concomitant treatments differed by the enalapril group being more frequently treated with thiazides (+6%) and less frequently with calcium channel blockers (−3%). Patients treated with enalapril had a generalized lower socio-economic status. There were no observed differences with regard to health care utilization (hospitalizations and length of stay, number of primary care visits and number of new diagnoses) between the two groups within 15 months from the start of the study. The proportion of included patients per year, from 1999 to 2007, showed a larger proportion of enalapril patients included at the end (2005–2007) of the observation period (data in Supplementary Table S3).

### Follow-up

The observation period comprised a total of 36 482 patient-years: 23 429 patient-years of enalapril treatment and 13 053 patient-years of candesartan treatment. The mean time (s.d.) of follow-up was 1.84 (1.97) years in the enalapril and 2.85 (2.31) years in the candesartan group.

There was no difference in the number of visits to primary care and laboratory/blood samples taken between the two groups during the first 2 years of the study (data in Supplementary Tables S4 And 5). Weight at baseline and weight during follow-up was similar in the groups (data in Supplementary Figure S1). During the observation period, 38.7% (*n*=4538) patients were discontinued from the enalapril-treated group and 27.1% (*n*=1157) from the candesartan group. Reasons for discontinuations were death: 2.6% (*n*=305) vs 2.5% (*n*=107), switch to other C09-drugs 20.0% (*n*=2345) vs 8.7% (*n*=372) or cessation of study drug prescription 16.1% (*n*=1888) vs 15.9% (*n*=678) in the enalapril group and the candesartan group, respectively.

### On-treatment blood pressures

**Figure 2:**
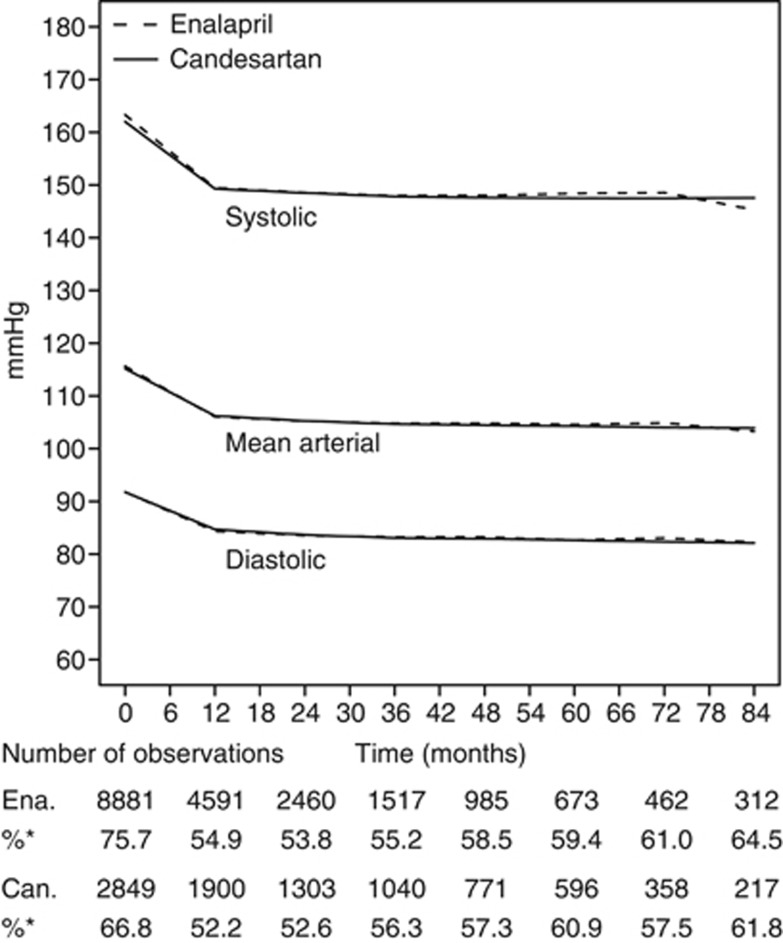
Blood pressure during follow-up. %*Percentage of blood pressure reading among patients at risk. Ena, enalapril; Can, candesartan.

The initiation of enalapril or candesartan was followed by a substantial blood pressure reduction, with no difference in blood pressure between the two treatment groups (Figure 2). The proportion of patients with blood pressure recordings was similar in both the treatment groups after 1 year of treatment.

### Incidence of new diagnosed diabetes

**Figure 3:**
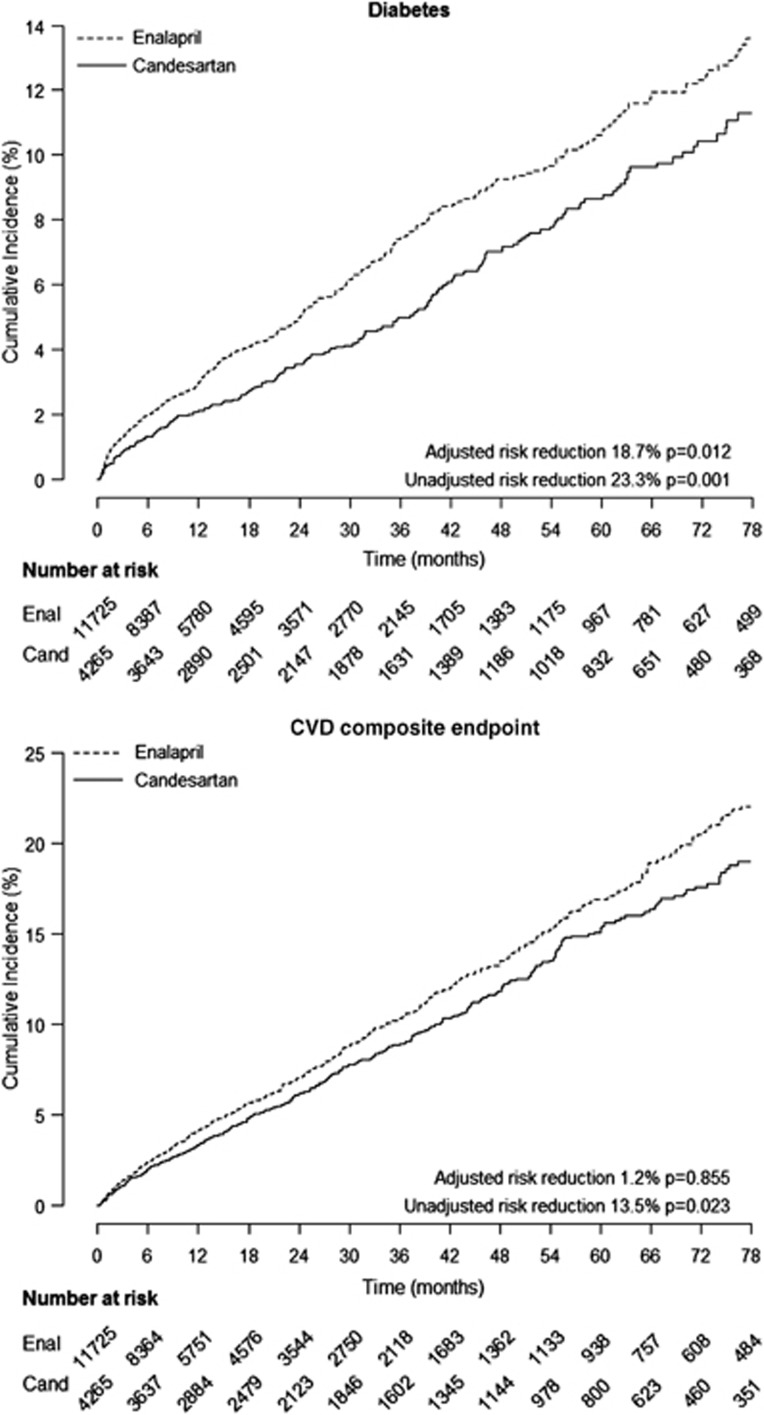
Kaplan–Meier curves for diabetes and composite CVD end point. Ena, enalapril; Can, candesartan.

A total of 991 subjects with a new diagnosis of diabetes were recorded during the observation period. The incidence rate was 0.074 per 100 patient-years and 0.066 per patient-years in the enalapril and candesartan group, respectively. The unadjusted risk of a new diagnosis of diabetes was lower (HR 0.77, 95% CI 0.66–0.90, *P*<0.01) in patients treated with candesartan compared with those with enalapril (Figure 3). This risk remained lower in candesartan patients after adjusting for age, gender, index year and socio-economic status, (HR 0.81, 95% CI 0.69–0.96, *P*=0.01).

Results of the additional sensitivity analyses with adjustments for baseline HbA1c, blood glucose and body mass index were consistent with the results from the main analysis for diabetes. The same result was also observed when diabetes diagnoses set within 6 and 12 months after the start of the study were excluded (Table 2). Few patients had high baseline HbA1c (>7% 0.14% vs 0.02%) or blood glucose (>7 mmol l^−1^, 3.99% vs 2.49% >10 mmol l^−1^, 0.37% vs 0.28%) values in the enalapril and candesartan groups. When these patients were excluded from the analyses, the results were also consistent with the main analysis (data in Supplementary Table S4).

The patient characteristics in the two groups after the propensity score matching are summarized in Table 1. In propensity score-matched analyses, candesartan patients had a lower risk of diabetes development, HR 0.63 (95% CI 0.42–0.96, *P*=0.03).

### Incidence of CVD

During the study, 785 CVD events occurred in the enalapril group and 375 in the candesartan group. The unadjusted risk of CVD was lower in candesartan patients than in enalapril patients (HR 0.87, 95% CI 0.76–0.98, *P*=0.02; Figure 3). When adjusting for covariates (age, gender, index year, socio-economic status), the risk was similar in the two groups (HR 0.99, 95% CI 0.87–1.13, *P*=0.86). Similar results were observed when multiple imputations were performed for systolic blood pressure.

In the 2222 patients in the propensity score-matched analysis, the HR of CVD was 0.83 (95% CI 0.56–1.24, *P*=0.37).

Additional sensitivity analyses were performed in order to explore the effect of variations in the proportion of included patients per year in addition to adjustment for inclusion year. The results with an adjusted HR of 1.00 (95% CI 0.87–1.15, *P*=1.00) for the cohort of included patients from 1999 until 2005 supported the main results.

### Treatment patterns

Both enalapril and candesartan were prescribed accordingly to the prescribing recommendations for hypertension. The enalapril group generally started with 5 mg (25.5%) or 10 mg (35.8%) and patients were up-titrated to 10 mg (31.0%) and 20 mg (36.5%) during the first 3 years of treatment. The use of fixed combination tablets (enalapril 20 mg/hydrochlorothiazide 12.5 mg) rose from 9.2% until 13.4% during the study period.

The candesartan group started mainly with 4 mg (27.6%) or 8 mg (50.3%) with an up-titration during the first 12 months of treatment to 43.2% for use of the 8 mg tablet and 20.4% for the 16 mg tablet. After 3 years of treatment, the patients treated with candesartan were mainly treated with 8 mg (35.3%), 16 mg (23.3%) or 16 mg/12.5 mg (24.1%) tablets.

The use of other antihypertensive medications increased in both groups during follow-up. Thiazides (both separate and in fixed combination tablets) were used more frequently in the enalapril group initially. This changed during follow-up; after 1 year on treatment, candesartan patients were more frequently treated with thiazides (34% vs 24%), and the difference in proportion of thiazides-treated patients between the two groups continued to increase during follow-up (data in Supplementary Figure S2). More calcium channel blockers and beta blockers were added in the enalapril group.

## Discussion

### Primary observations

In this comparative effectiveness study of 15 990 hypertension patients without CVD or diabetes in real-life primary care, initiation of enalapril or candesartan was followed by a substantial blood pressure reduction, with no difference in blood pressure between the two treatment groups during the follow-up period. Candesartan patients had, however, a lower risk of new diagnosed diabetes compared with enalapril patients. These results were consistent across different analyses and subpopulations (data in Supplementary Figure S3). No difference in CVD risk was observed between the two groups.

### Interpretation with reference to other studies

The results of this study suggest that there is a risk reduction of new-onset diabetes with candesartan compared with enalapril in the treatment of hypertension. Both ACEi and ARBs have in previous studies shown a reduction in new onset of diabetes.^1, 2, 3, 4^ A reduction in new onset of diabetes in the ARB group compared with the ACEi group may be supported by previous observations.^5^ Lack of activation of parts of the PPAR system with ACEi treatment, and thus less stimulation of glucose activation, has been postulated as an explanation for potential differences vs ARB in the prevention of new onset of diabetes.^6^ Candesartan has a tight and long-lasting binding to the AT type 1 receptor.^23^ The potential to prevent new-onset diabetes may therefore be explained by a more effective blockade of AT type I receptors and the subsequent development of vascular insulin resistance and impaired endothelial nitric oxide-mediated relaxation.^7^

During the study, there was no difference between the two treatments in protection for CVD. This finding is in line with results from randomized controlled studies comparing the CVD-protective effect of ACEi and ARB treatments.^8, 9^ Differences with regard to new-onset diabetes rates during the study may not be expected to affect CVD incidence due to the relatively short study duration. The treatment period for patients treated with enalapril was generally shorter, indicating a lower tolerability for enalapril compared with candesartan. These findings, indicating a lower tolerability of ACEi treatment, are in agreement with findings from other real-life and randomized controlled studies.^23, 24, 25, 26, 27^

### Strengths and limitations

The present study was performed using primary care data from primary care centers which represented 7% of all primary care centers in Sweden. High-quality national data on hospitalizations, prescribed drugs and causes of death were also included. This provides a representative selection of patients and a more or less complete long-term follow-up of newly diagnosed diabetes and major cardiovascular events.

### Potential effect of unmeasured confounders

As commonly in non-randomized studies of the effectiveness of drug treatment, it cannot be excluded that residual confound may have influenced the findings. In-depth understanding for why physicians choose enalapril or candesartan for treatment for hypertension can only be explored by quality interviews with the prescribing physicians, data we unfortunately do not have access to in this study. Data on smoking and physical activity were missing for the majority of patients and was therefore not included in the analyses. The general socio-economic status was lower in the enalapril group, and potentially more patients could be expected to smoke in this group or have a different physical activity profile. The difference in socio-economic status is, however, adjusted for in all the analyses. We did not observe a difference between the two groups with regard to the proportions of patients with chronic obstructive pulmonary disease and or use of chronic obstructive pulmonary disease medications, which is closely related to smoking. Nor did we see differences in mean weight during follow-up (data in Supplementary Figure S3). In consideration of the possible impact of residual confounding, it should be recognized that Sweden has a tax-funded healthcare system with equal access to health-care services and drugs, thus choice of treatment and patient follow-up should be primarily based on clinical data and not on non-medical reasons. We did not observe differences in how the patients were treated and followed up before and after the start of study medication in the recorded data.

### Missing blood pressure values

One of the limitations with this method is missing data in the electronic patient primary care journals. Blood pressure recordings were registered in 72% of all the patients at baseline. The enalapril group had a slightly higher baseline systolic blood pressure compared with the candesartan group. However, analysis with multiple imputations for missing systolic blood pressure and analysis with adjustment for available systolic blood pressures gave the same results (Table 2).

### Opportunistic diagnosis

A potential explanation of the finding of more new diagnoses of diabetes in the enalapril group could be ‘opportunistic diagnosis' due to a potential increased number of patient visits to primary care in this group who had a higher non-CVD burden. However, the frequency of primary care visits, diagnoses, laboratory/blood samples data and hospitalizations before the start of the study did not differ markedly between the two groups, suggesting similar needs for medical consultations at baseline. We did not observe any major difference in the number of annual primary care visits or blood samples taken between the two treatment groups during follow-up (data in Supplementary Tables S5 and S6). The finding of increased number of diabetes diagnoses in the enalapril group did not follow the general trend regarding other diagnoses during the observation period as the number of other diagnoses made during the study was higher in the candesartan group. This does not support the possibility of a general higher disease burden in the enalapril group (data in Supplementary Table S7).

### Risk of the differential exclusion of patients

Enalapril and candesartan have the same prescribing indications in Sweden; both are indicated for hypertension and heart failure but not for renal diseases. However, the ACEis were developed before the ARB class and thus gained hard end point documentation and CVD indications (heart failure, myocardial infarction) earlier. More patients (11.2%) were excluded for earlier diabetes and CVD in the enalapril group. Patient records in primary care were searched for chronic kidney disease, diabetes and CVD diagnoses and drugs up to 5–6 years before inclusion. The same diagnoses were also searched for in the National Patient Register, which has a national coverage since 1987.^12^ The combination of these two search techniques should therefore have lowered the risk of undetected diabetes and CVD prevalence at baseline.

### Difference in treatment practice over time

When including patients over a long time span, an important potential confounding factor could have been variations in hypertensive treatment over time, favoring inclusion either in the enalapril or candesartan group. Alterations in the Swedish reimbursement system for the use of RAAS (renin–angiotensin–aldosterone system)-inhibiting drugs for hypertension in 2008 are an example. Qualifications for reimbursement for hypertension from this date required that patients should start with an ACEi and ARBs should be prescribed as a second-line treatment for patients with side effects on ACEi treatment or as an add-on therapy (heart failure). These requirements were implemented earlier in some areas of Sweden. The annual frequency of inclusion to the enalapril or candesartan group from 1999 to 2007 reflects these changes; by a relatively higher use of enalapril from 2005 (data in Supplementary Table S3). In order to to minimize the possible effects of temporal changes, index year (start of treatment) was included as covariate/adjustment in all the analyses. The same results were observed when we excluded patients included in 2005–2007 from the study.

The study had a follow-up time of mean (s.d.) 2.11 (2.11) years. There was a major difference in follow-up time between the two groups, the enalapril group with a mean (s.d.) of 1.84 (1.97) years and a mean 2.85 (2.31) years in the candesartan group. This difference can partly be explained by a larger portion of enalapril patients included at the end of the observation period. Nevertheless, when excluding patients included during the last 3 years of the observation period, the enalapril patients still have, in general, a shorter median follow-up period (−0.84 years) caused by higher number of patients who switched to other C 09 drugs or ending their enalapril treatment.

### Perspectives

Our study method can be used to study existing treatments, providing results faster than performing a prospective randomized clinical trial and at a moderate cost. Sweden offers the unique combination of a wide use of similar electronic patient record systems in primary care and a long tradition with nationwide hospitalization and cause of death registers. This provides the unique opportunity to study differences between treatments, which are not possible to assess in randomized clinical trials.

The results of this study suggest that there is a risk reduction of new-onset diabetes with candesartan compared with enalapril in the primary treatment of hypertension, while the two treatments provide similar protection for CVD. Patients treated with enalapril had a shorter treatment period, indicating a lower tolerability for enalapril compared with candesartan. The results of this retrospective study should be confirmed, however, in prospective studies before any definitive conclusions are made.

## Figures and Tables

**Table 1:** Baseline data from 15 990 hypertensive patients without previous cardiovascular disease and diabetes

*Variable*	*Unmatched*			*Propensity score matched*		
	*Enalapril (*n*=11 725)*	*Candesartan (*n*=4265)*	P*-value*	*Enalapril (*n*=1111)*	*Candesartan (*n*=1111)*	P*-value*
Age (years)	61.0 (12.1)	60.0 (11.6)	<0.01	59.6 (10.8)	59.7 (10.7)	0.81
Women, *n* (%)	6216 (53)	2431 (57)	<0.01	582 (52)	583 (53)	1.00
Body mass index (kg* *m^−2^)	29.2 (5.3)	28.9 (5.2)	0.10	28.8 (4.8)	29.5 (5.2)	0.04
Systolic blood pressure (mm* *Hg)	163.3 (19.1)	162.0 (19.2)	<0.01	161.5 (18.7)	161.7 (18.3)	0.80
Diastolic blood pressure (mm* *Hg)	91.8 (10.6)	91.8 (10.4)	0.94	92.2 (10.2)	92.1 (10.2)	0.71
Total cholesterol (mmol* *l^−1^)	5.9 (1.0)	5.8 (1.0)	0.11	5.9 (1.0)	5.9 (1.0)	0.88
LDL cholesterol (mmol* *l^−1^)	3.6 (0.8)	3.6 (0.8)	0.90	3.6 (0.8)	3.6 (0.8)	0.79
HDL cholesterol (mmol* *l^−1^)	1.4 (0.3)	1.4 (0.3)	0.92	1.4 (0.3)	1.3 (0.3)	<0.01
Triglycerides (mmol* *l^−1^)	1.6 (0.8)	1.6 (0.8)	0.37	1.6 (0.7)	1.7 (0.8)	0.12
Glucose (mmol* *l^−1^)	5.4 (1.1)	5.3 (1.1)	<0.01	5.3 (1.3)	5.3 (1.3)	0.63
HbA1c (%)	4.9 (0.7)	4.7 (0.5)	<0.01	4.7 (0.5)	4.9 (0.7)	<0.01
Serum creatinine (μmol* *l^−1^)	79.6 (16.7)	82.3 (16.2)	<0.01	81.4 (16.1)	82.0 (16.2)	0.41
Potassium (mmol* *l^−1^)	4.1 (0.3)	4.1 (0.3)	0.12	4.1 (0.3)	4.1 (0.3)	0.57
Thiazides, *n* (%)	2082 (18)	525 (12)	<0.01	204 (18)	197 (18)	0.74
Calcium channel blockers^a^, *n* (%)	1181 (10)	555 (13)	<0.01	172 (15)	181 (16)	0.64
Beta blockers, *n* (%)	2855 (24)	1050 (25)	0.74	351 (32)	366 (33)	0.52
Statins, *n* (%)	749 (6)	290 (7)	0.37	137 (12)	137 (12)	0.95
Socio-economic status^b^ (low/medium/high)	35/33/32	31/32/37	<0.01	33/29/39	32/30/38	0.76
Percentage of patients hospitalized for any reason^c^	10.6%	11.1%				
Number of visits in primary care^c^	2.0	2.0				
Total number of diagnoses set (100 patients year^−1^)^c^	196.3	196.7				

Abbreviations: HbA1c, hemoglobin A1c; HDL, high-density lipoprotein; LDL, low-density lipoprotein.

The numbers in brackets represents s.d., where no other description is given.

a Dihydropyridine substances.

b Educational level.

c Within 15 months before the start of study.

**Table 2:** Effect of additional adjustment and different analysis methods on clinical outcomes obtained from primary care journals and Swedish national discharge and death registers

	*Number of patients*			
	*Enalapril,* n	*Candesartan,* n	*HR, new-onset diabetes*	*HR, CVD*
Unadjusted	11 725	4265	0.77 (95% CI 0.66–0.90)	0.87 (95% CI 0.76–0.98)
				
Primary adjusted results^a^	11 725	4265	0.81 (95% CI 0.69–0.96)	0.99 (95% CI 0.87–1.13)
+systolic BP (Multiple imputed values)^b^	11 725	4265	0.80 (95% CI 0.68–0.94)	0.92 (95% CI 0.81–1.05)
+systolic BP (available values)^b^	8881	2849	0.79 (95% CI 0.65–0.96)	0.97 (95% CI 0.83–1.13)
+HbA1c^b^	1151	428	0.79 (95% CI 0.58–1.07)	−
+blood glucose^b^	7338	2256	0.78 (95% CI 0.64–0.96)	−
+BMI^b^	2896	772	0.86 (95% CI 0.97–1.15)	−
				
Excluding patients diagnosed within 6 months after the start of the study^c^	11 520	4212	0.87 (95% CI 0.72–1.05)	0.98 (95% CI 0.86–1.12)
Excluding patients diagnosed within 12 months after the start of the study^c^	11 443	4185	0.88 (95% CI 0.72–1.10)	0.97 (95% CI 0.85–1.11)
Propensity score analysis^d^	1111	1111	0.63 (95% CI 0.42–0.96)	0.88 (95% CI 0.56–1.24)

Abbreviations: BMI, body mass index; BP, blood pressure; CVD, cardiovascular disease; HbA1c, hemoglobin A1c; HR, hazard ratio.

a Adjusted for age, gender, index year and socio-economic status.

b Added adjustments to primary adjustments.

c Primary adjustments.

d Matched for gender, age, index year, systolic blood pressure, total cholesterol, blood glucose, socio-economic status, beta blockers, statins, calcium antagonists and thiazides.

## Notes

LPH is enrolled at the PhD School of the University of Oslo, Faculty of Medicine. LPH, NH and JB are employed by AstraZeneca. AstraZeneca sells candesartan in many markets. Candesartan has lost patent protection. MT is employed by an independent statistical consultant company, Statisticon, for which AZ is a client. J Stålhammar has received honoraria for lecturing/consulting and other research support from AZ. J Sundström has received honoraria for advisory board participation from AZ. DR has received honoraria for lecturing/consulting and other research support from AZ, Boehringer Ingelheim, Pfizer and Bayer. SEK has received honoraria for lecturing/consulting and other research support from AZ, Bayer, Medtronic, MSD, Pronova, Serodus and Takeda.
