# Supplementary material for: Diabetes and CVD risk during angiotensin-converting enzyme inhibitor or angiotensin II receptor blocker treatment in hypertension: a study of 15 990 patients
Source: J Hum Hypertens. 2014 Jun 26;28(11):663–9. doi: 10.1038/jhh.2014.43 (PMC4191159; doi:10.1038/jhh.2014.43)
Supplement: Supplementary Information [file jhh201443x1.doc]

**ONLINE SUPPLEMENT**

Diabetes and CVD risk during angiotensin converting enzyme inhibitor or angiotensin II receptor blocker treatment in hypertension: a study of 15 990 patients

Authors: Pål Hasvold M Sc Pharm (1), Johan Bodegård MD PhD (2), Marcus Thuresson PhD (3), Jan Stålhammar MD (4), Niklas Hammar (5) PhD, Johan Sundström MD PhD (6), David Russell MD PhD (7), Sverre E. Kjeldsen MD PhD (8)

Affiliations:

1. University of Oslo, Oslo, Norway and AstraZeneca
2. Department of Cardiology, Ullevaal hospital, Oslo, Norway and AstraZeneca
3. Statisticon AB, Uppsala, Sweden
4. Department of Public Health and Caring Sciences, Uppsala University, Uppsala, Sweden
5. Institute of Environmental Medicine, Karolinska Institutet, Stockholm, Sweden and AstraZeneca R&D, Mölndal
6. Department of Medical Sciences & Uppsala Clinical Research Center, Uppsala University, Uppsala, Sweden
7. Department of Neurology, Rikshospitalet, University of Oslo, Oslo, Norway
8. Department of Cardiology, Ullevaal Hospital, University of Oslo, Oslo, Norway

Correspondence: Pål Hasvold

AstraZeneca Nordic-Baltic

Medical department

Postboks 6050 Etterstad

N-0601 Oslo, Norway

E-mail: paal.hasvold@astrazeneca.com

Fax: 0047 23294145

Phone: 0047 21006400

Expanded Methods and Results

Table S1: Diagnoses for excluding patients and registration of endpoints. The current diagnosis had to be present in primary care journals or in hospital data prior study start to excluded patients or present after study start in order to be recorded as an endpoint.

|  | ICD-10 | ICD-9 |
| --- | --- | --- |
| **Cardiovascular Disease** |  |  |
| Heart failure | I50, I11.0 | 428 |
| Cardiac arrhythmias | I46–I48 | 427 |
| Peripheral artery disease | I70, I71, I74 | 440, 441, 444 |
| Chronic ischemic heart disease | I20.9, I25.1 | 413–414 |
| Unstable angina pectoris | I20.0 | 411 |
| Myocardial infarction | I21–I23 | 410, 411, 429 |
| Stroke | I61, I63–I64, G45 | 431–434, 435 |
| **Chronic kidney disease** | N08.3, N18, N19 | 585.1-585.6 |
| **Diabetes** | E10–E14 | 250 |
|  | and or use of anti-diabetic drugs (ATC: A10) | |

Table S2: Selection of study centers

| 29 (40%) | Small clinics, 1-4 physicians |
| --- | --- |
| 33 (47%) | Midsize clinics, 5-10 physicians |
| 9 (13%) | Large clinics, >10 physicians |

The final selection of centers included 54 public primary care centers and 26 private care centers. With rural areas defined as communities with no more than 15 000 inhabitants, 35 of the sites were considered rural and 36 sites were defined as urban.

Table S3: Included subjects summarized by year of inclusion

|  | Enalapril (N=11 725) | Candesartan (N=4 265) | Total (N=15 990) |
| --- | --- | --- | --- |
| 1999 | 656 (5.6) | 274 (6.4) | 930 (5.8) |
| 2000 | 543 (4.6) | 302 (7.1) | 845 (5.3) |
| 2001 | 626 (5.3) | 474 (11.1) | 1100 (6.9) |
| 2002 | 738 (6.3) | 586 (13.7) | 1324 (8.3) |
| 2003 | 734 (6.3) | 483 (11.3) | 1217 (7.6) |
| 2004 | 1132 (9.7) | 493 (11.6) | 1625 (10.2) |
| 2005 | 2083 (17.8) | 436 (10.2) | 2519 (15.8) |
| 2006 | 2460 (21.0) | 563 (13.2) | 3023 (18.9) |
| 2007 | 2753 (23.5) | 654 (15.3) | 3407 (21.3) |

Table S4: Outcome for diabetes when excluding patients with elevated HbA1c or blood glucose at baseline

|  | **Enalapril** | **Candesartan** | **Hazard ratio**  **(adjusted)** | **95 % Confidence**  **Interval** | **p-value** | **Hazard ratio**  **(unadjusted)** | **95 % Confidence**  **Interval** | **p-value** |
| --- | --- | --- | --- | --- | --- | --- | --- | --- |
| **Excluding patients with HbA1c >7** | n =11 709 | n =4 264 |  |  |  |  |  |  |
|  | 521 (4.4) | 220 (5.2) | 0.83 | (0.71- 0.98) | 0.03 | 0.79 | (0.67- 0.92) | <0.01 |
| **Excluding patients with blood glucose >7** | n = 11 257 | n = 4 159 |  |  |  |  |  |  |
|  | 369 (3.3) | 183 (4.4) | 0.90 | (0.75- 1.08) | 0.26 | 0.86 | (0.721- 1.031) | 0.10 |
| **Excluding patients with blood glucose >10** | n = 11 682 | n = 4 253 |  |  |  |  |  |  |
|  | 503 (4.3) | 213 (5) | 0.83 | (0.70- 0.97) | 0.02 | 0.78 | (0.664- 0.918) | <0.01 |

Table S5: Mean number of visits to primary care after study start

|  | Year 1 | Year 2 | Year 3 |
| --- | --- | --- | --- |
| Enalapril | 3.7 | 3.7 | 3.8 |
| Candesartan | 3.7 | 3.7 | 3.7 |

Table S6: % of patients with laboratory measurement after study start

|  | **Haemoglobin**  (% of patients with measurement) | | | **Blood glucose**  (% of patients with measurement) | | |
| --- | --- | --- | --- | --- | --- | --- |
|  | Year 1 | Year 2 | Year 3 | Year 1 | Year 2 | Year 3 |
| Enalapril | 48.7 | 51.1 | 52.0 | 42.9 | 45.7 | 45.0 |
| Candesartan | 49.0 | 48.4 | 51.3 | 40.9 | 42.1 | 43.4 |

Table S7: New diagnoses recorded after study start

|  | New diagnoses/100 patients/  year set during follow up |
| --- | --- |
| Enalapril | 178.6 |
| Candesartan | 186.6 |

Figure S1: Mean patient weight during follow up


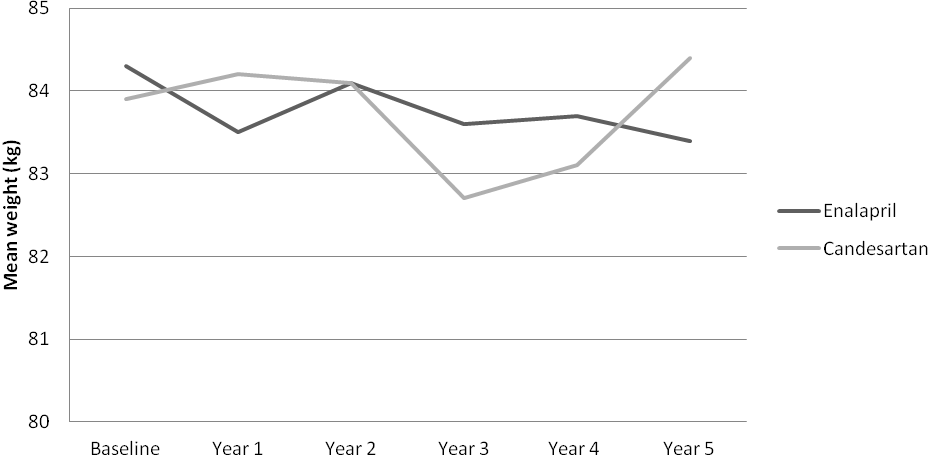


Figure S2: Concomitant use of thiazides during follow up


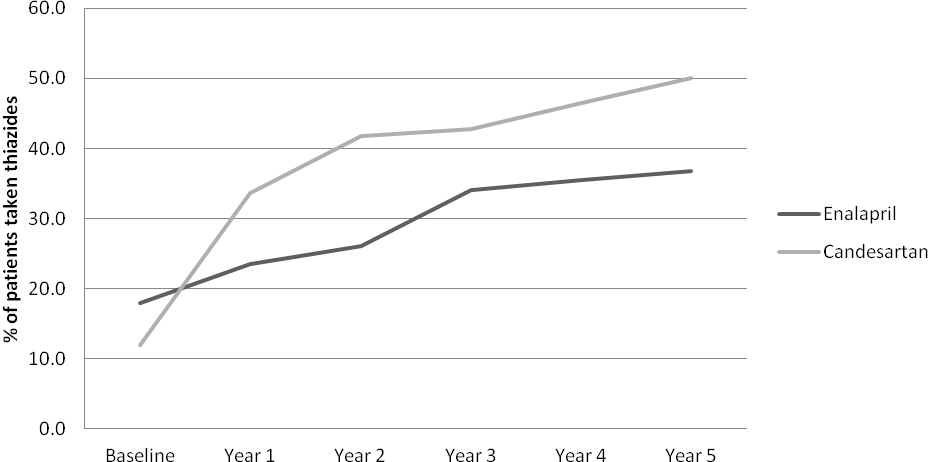


Figure S3: Time to new onset of diabetes in the study population and in subgroups

|  | Candesartan | | Enalapril | |  |  |  |  |  |  |  |  |  |  |  |  |  |  |  |  |  |  |  |  |  |  |  |  |  |
| --- | --- | --- | --- | --- | --- | --- | --- | --- | --- | --- | --- | --- | --- | --- | --- | --- | --- | --- | --- | --- | --- | --- | --- | --- | --- | --- | --- | --- | --- |
|  | n/N | Events/1 000  patient years | n/N | Events/1 000  patient years | Hazard ratio  (95 % CI) | P-value | P-value  for interaction |  |  |  |  |  |  |  |  |  |  |  |  |  |  |  |  |  |  |  |  |  |  |
| All patients | 220/4 265 | 18.9 | 536/11 725 | 26.0 | 0.81 (0.69:0.96) | 0.01 |  |  |  |  |  |  |  |  |  |  |  |  |  |  |  |  |  |  |  |  |  |  |  |
| Previously untreated* | 134/2 790 | 18.1 | 381/7 570 | 27.7 | 0.74 (0.60:0.90) | <0.01 | 0.05 |  |  |  |  |  |  |  |  |  |  |  |  |  |  |  |  |  |  |  |  |  |  |
| Previously treated^†^ | 86/1 475 | 20.3 | 155/4 155 | 22.7 | 0.98 (0.74:1.30) | 0.91 |  |  |  |  |  |  |  |  |  |  |  |  |  |  |  |  |  |  |  |  |  |  |  |
| Men | 122/1 834 | 25.3 | 308/5 509 | 30.8 | 0.88 (0.71:1.09) | 0.23 | 0.28 |  |  |  |  |  |  |  |  |  |  |  |  |  |  |  |  |  |  |  |  |  |  |
| Women | 98/2 431 | 14.4 | 228/6 216 | 21.5 | 0.74 (0.58:0.94) | 0.02 |  |  |  |  |  |  |  |  |  |  |  |  |  |  |  |  |  |  |  |  |  |  |  |
| >=65 years | 62/1 399 | 16.5 | 195/4 506 | 25.2 | 0.79 (0.58:1.07) | 0.12 | 0.54 |  |  |  |  |  |  |  |  |  |  |  |  |  |  |  |  |  |  |  |  |  |  |
| <65 years | 158/2 866 | 20.1 | 341/7 219 | 26.5 | 0.82 (0.68:1.00) | 0.05 |  |  |  |  |  |  |  |  |  |  |  |  |  |  |  |  |  |  |  |  |  |  |  |
| High sys BP | 69/1 382 | 16.7 | 226/4 455 | 30.1 | 0.61 (0.46:0.81) | <0.01 | 0.01 |  |  |  |  |  |  |  |  |  |  |  |  |  |  |  |  |  |  |  |  |  |  |
| Low sys BP | 73/1 467 | 19.1 | 152/4 426 | 20.5 | 1.05 (0.79:1.40) | 0.74 |  |  |  |  |  |  |  |  |  |  |  |  |  |  |  |  |  |  |  |  |  |  |  |
| High HbA1c | 51/189 | 132.0 | 166/577 | 227.5 | 0.67 (0.48:0.93) | 0.02 | 0.85 |  |  |  |  |  |  |  |  |  |  |  |  |  |  |  |  |  |  |  |  |  |  |
| Low HbA1c | 10/250 | 13.7 | 21/604 | 23.9 | 0.45 (0.20:1.00) | 0.05 |  |  |  |  |  |  |  |  |  |  |  |  |  |  |  |  |  |  |  |  |  |  |  |
| High BMI | 49/382 | 44.9 | 140/1 445 | 58.7 | 0.88 (0.63:1.23) | 0.44 | 0.31 |  |  |  |  |  |  |  |  |  |  |  |  |  |  |  |  |  |  |  |  |  |  |
| low BMI | 16/390 | 15.2 | 65/1 451 | 26.6 | 0.63 (0.36:1.11) | 0.11 |  |  |  |  |  |  |  |  |  |  |  |  |  |  |  |  |  |  |  |  |  |  |  |
| Low socioeconomic status | 82/1 290 | 21.9 | 183/4 005 | 24.2 | 0.97 (0.74:1.26) | 0.81 |  |  |  |  |  |  |  |  |  |  |  |  |  |  |  |  |  |  |  |  |  |  |  |
| Medium socioeconomic status | 66/1 318 | 18.2 | 190/3 762 | 29.7 | 0.69 (0.52:0.92) | 0.01 | 0.07 |  |  |  |  |  |  |  |  |  |  |  |  |  |  |  |  |  |  |  |  |  |  |
| High socioeconomic status | 68/1 557 | 16.9 | 142/3 685 | 23.3 | 0.79 (0.59:1.06) | 0.12 | 0.31 |  |  |  |  |  |  |  |  |  |  |  |  |  |  |  |  |  |  |  |  |  |  |

*Patients with no prior treatment for high blood pressure at start of study

^†^Patients with ongoing treatment for high blood pressure at start of study
